# Case of Purulent Pleurisy

**Published:** 1870-08

**Authors:** John W. H. Baker

**Affiliations:** Davenport, Iowa


ART. VIII.—*Case of Purulent Pleurisy.* By JOHN W. H. BAKER, M.D., Davenport, Iowa. Read before the Iowa State Medical Society, Feb 24th, 1870.

We seldom find more interesting cases than those frequently occurring and usually denominated pleuritic effusions. These cases most commonly arise from acute pleuritis, or the associated disease, pleuro-pneumonitis, and such cases are rendered more positively interesting in tracing its progress either to recovery or death. In this connection I report the following case:

On the 4th day of September last, I was called to visit Otto S——, a young man of a robust, healthy habit, about 21 years of age. He had always enjoyed uninterrupted good health. Had been accustomed to go on hunting excursions upon the river, and about the sloughs and islands below the city. Had been on one of these hunting excursions a few days previously, and became wet and cold. He thus remained out upon one of the islands over night, sleeping in an exposed open space. On his return he was aware that he had taken cold, and the result was an attack of most violent pain on the left side, in the cardiac region. When I arrived I found him suffering with sharp, cutting and lancinating pains, with short checked respiratory efforts, marked febrile excitement. The pains were much increased on effort to get a full inspiration. Skin was dry and hot. Tongue moderately coated. Pulse full, frequent and incompressible. Associated with these symptoms were intense thirst and torpor of the bowels. Put the patient under treatment; gave active cathartics, applied counter-irritants and heat; gave an opiate after the action of the cathartics, and endeavored to promote perspiration. Inflammatory symptoms were quite persistent, and the pain and prostration of the most marked character, indicative of acute pleurisy for the period of ten days or thereabout. After the subsidence of the severe pains, the thoracic walls upon the left side, in the region of the pain, showed a bulging or prominence over the cardiac region. Upon percussion, the locality gave an extended dull sound, while auscultation evinced a masked sound of the heart. The action of the heart was violent; so much so as to create a cardiac tremor over the whole left side. Respiration was impeded on the left, and in all the lower lobe was destitute of the ordinary vesicular murmur. Upon succussion, the manifest presence of liquid could not only be felt but was easily heard, even by persons sitting by the side of the bed, giving the sound of liquid and air confined in the same cavity. Counter-irritants and absorbent remedies, cathartic and diuretic medicines, were tried for the next ten days, when the increased prominence of the left side, and the marked protrusion between the ribs, indicated the necessity of puncture. During all this time the patient had suffered intensely, being greatly emaciated, and presenting an almost hopeless condition.

On the 24th of September, twenty days from the commencement of the attack, being fully convinced that the only hope for the patient (and that a small one) was by puncture, I called in counsel, and, as the result of advice, punctured at a point two inches in a direct line below the left nipple, that point indicating a thinning of the thoracic walls, and an approach of liquid contents to the surface. With a sharp-pointed bistoury, the incision was made through the skin and cellular substance, which opened directly into the cavity containing air and purulent matter. The patient lying directly on his back at the time of puncture, rendered the first issue of material from the cavity necessarily air or gas of the most fetid odor. After the air had escaped, as from a compressed sac, with a hissing noise, the prominence was somewhat diminished, and we turned the patient partly on his left side, when a very consistent and terribly odious pus was caused to flow from the punctured orifice; occasionally the incision would become blocked by thickened masses of shreddy pus, when by means of the probe they would be broken down, and the flow established again. About three pounds of matter were in this way removed. We then dressed the wound with plaster and compress, and the patient acknowledged that he felt some relief. For the next ten or twelve days we removed each day from a half pound to one or two pounds of pus, being careful that the opening was as well guarded from the entrance of air as possible. About this time, finding the issue of matter to be about the same from day to day, and the evidence of a large amount of purulent matter still retained in the thoracic cavity, and having acted on the cautious plan of evacuating only a portion of the contents at each time, we concluded to try a means of more completely emptying the cavity, and for this purpose arranged a medium sized catheter, so as to form, with some rubber tubing, a syphon. To the rubber tube we attached a rubber suction ball. With this apparatus we introduced the catheter end into the thoracic cavity, passing it some six inches into the liquid contents, then with the air expelled from the suction ball, we attached it to the rubber end, which thus exhausting the tube, caused the purulent matter to flow in a continuous stream. The, tube occasionally blocking up, with the ball we would force the flow again. In this way, at this time, we removed five pounds of thick offensive pus from the thoracic cavity. This operation was repeated daily, obtaining on one other day six pounds, and subsequently from half a pound to a pound of matter each day. We made memoranda of the amount removed until it amounted to over thirty pounds: meanwhile we were giving the patient Iron and tonic remedies, and such treatment as the symptoms from time to time indicated, the case necessarily requiring a variable treatment. For instance, during a certain stage of this sickness, the patient was attacked with dysentery, and required active remedies to control the dysenteric stools, which were frequent, bloody and painful. At another time, he had indications of a positive pneumonitis, and required remedies for the complication. But, aside from these complications, the general indications were for a tonic course, with nutritious diet, and anything tending to support the strength and nourish and support the waste of tissue, so actively in progress, evidenced in the discharge of such large quantities of purulent matter.

Continuing our efforts to exhaust this fountain of pus, we were considerably troubled with the blocking up of our tube, and finally tried a variety of means, such as the attachment of an air pump or stomach pump, to force the obstructing mass through the tube, but without success. The quantity continued about the same (about eight ounces) each day. We then obtained a very strong ball from an old fashioned ball breast pump, and in the place of our smaller and weaker suction ball, used this. Still our tube would occasionally block up or become obstructed. We finally concluded to withdraw the tube whenever it became so obstructed, and by so doing we discovered that the obstructing matter consisted of floating masses of shreddy, fibrinous tissue, which being caught in the eye or opening of the catheter, could be brought to the orifice or puncture, when, by great care, they were seized with a small pair of spring forceps, and carefully removed. In this way, on one occasion, we removed about eight ounces of this membranous floating material. After the removal of this quantity of shreds, the character of the matter removed was less offensive, and very gradually lessened in quantity. The cavity was evidently diminishing in its capacity, for we now began the use of injections of a solution of carbolic acid. Our first injections were of about one pound of liquid of the strength of ten grains of acid to the ounce. We injected with this fluid about once each week, gradually increasing the strength, obtaining good evidence of amendment after each injection. A gradual improvement of the respiration occurred, keeping pace with the lessening of the discharge. The superior portion of the left lung became more active, the right lung quieting down to a more natural action, having previously performed almost the whole function of both lungs.

The amount of matter drawn from this cavity lessened from time to time, until after five months and about ten days the formation of matter wholly ceased. The general health and fleshy condition of this patient has been fully re-established. He is now able to do moderate work, and looks robust.

This case is interesting, from the severe and very marked symptoms in the commencement of an acute pleurisy; the rapid progress made to a stage in which effusion took place; the large amount of effusion, and the peculiar prominence of the thoracic walls; the approach of the liquid contents toward the surface and final presentment at the peculiar point where opened. Finally, it is interesting in the extremely large amount (over fifty pounds) of thick cream-like pus and shreddy formations which were removed; and what is most remarkable is, that the patient should have recovered from a disease so generally fatal in its results.

We claim that the method, together with the continued and persevering removal of the purulent accumulation, contributed largely to bring about so desirable a result, while we acknowledge a strong, robust and healthy constitution as a helping handmaid in this case.

